# A Novel Microtubule Inhibitor 4SC-207 with Anti-Proliferative Activity in Taxane-Resistant Cells

**DOI:** 10.1371/journal.pone.0079594

**Published:** 2013-11-06

**Authors:** Elena Bausch, Hella Kohlhof, Svetlana Hamm, Rolf Krauss, Roland Baumgartner, Lucia Sironi

**Affiliations:** 1 Department of Biology and Konstanz Research School Chemical Biology, University of Konstanz, Konstanz, Germany; 2 4SC AG, Planegg - Martinsried, Germany; 3 4SC Discovery GmbH, Planegg, Martinsried, Germany; Virginia Tech, United States of America

## Abstract

Microtubule inhibitors are invaluable tools in cancer chemotherapy: taxanes and vinca alkaloids have been successfully used in the clinic over the past thirty years against a broad range of tumors. However, two factors have limited the effectiveness of microtubule inhibitors: toxicity and resistance. In particular, the latter is highly unpredictable, variable from patient to patient and is believed to be the cause of treatment failure in most cases of metastatic cancers. For these reasons, there is an increasing demand for new microtubule inhibitors that can overcome resistance mechanisms and that, at the same time, have reduced side effects. Here we present a novel microtubule inhibitor, 4SC-207, which shows strong anti-proliferative activity in a large panel of tumor cell lines with an average GI_50_ of 11nM. In particular, 4SC-207 is active in multi-drug resistant cell lines, such as HCT-15 and ACHN, suggesting that it is a poor substrate for drug efflux pumps. 4SC-207 inhibits microtubule growth *in vitro* and *in vivo* and promotes, in a dose dependent manner, a mitotic delay/arrest, followed by apoptosis or aberrant divisions due to chromosome alignment defects and formation of multi-polar spindles. Furthermore, preliminary data from preclinical studies suggest low propensity towards bone marrow toxicities at concentrations that inhibit tumor growth in paclitaxel-resistant xenograft models. In summary, our results suggest that 4SC-207 may be a potential anti-cancer agent.

## Introduction

Microtubule inhibitors (MTIs) have been very successful in cancer therapy against a number of tumors: taxanes are commonly used in the treatment of breast and ovarian cancers while vinca alkaloids have been very effective in the treatment of hematological malignancies [1].

Unfortunately, the efficacy of MTIs has been limited on one hand by toxicities, e.g. neutropenia and peripheral neuropathies [2,3], and on the other hand by the development of drug resistance [4,5]. While side effects are well understood and are usually manageable by dose reduction and/or dose interval, drug resistance poses problems to long-term usage of MTIs and has been estimated to be the cause for treatment failure in >90% of patients with metastatic disease [6]. 

MTIs bind to soluble or polymerized tubulin and, by doing so, affect microtubule dynamics [1]. Microtubules are polymers composed of α,β tubulin dimers that can exist in a growing or a shrinking phase. This dynamic behavior allows microtubules to fulfill most of their functions that range from intracellular transport to cell shape maintenance, from cell polarity to cell signaling and cell division [7,8].

MTIs are classified into two main groups: microtubule-stabilizing agents (such as taxanes and epothilones), which stimulate microtubule polymerization, and microtubule-destabilizing agents (such as vinca alkaloids, colchicine and halichondrins), which inhibit microtubule polymerization [5]. This classification holds true at high MTI concentrations, while at 10 to 100-fold lower concentrations both classes are known to suppress microtubule dynamics without affecting the amount of total polymer mass [9,10].

In mitosis, where dynamic microtubules are crucial for proper spindle function, suppression of microtubule dynamics will inhibit the correct assembly of the mitotic spindle, activate the spindle assembly checkpoint and subsequently promote mitotic delay or arrest followed either by aberrant divisions or cell death [11,12]. 

Even though the mechanism by which MTIs promote mitotic arrest is well understood, relatively little is known about how MTIs act in the context of a tumor and why drug sensitivity varies amongst different cancers, i.e. why taxol is effective against ovarian and mammary tumors but is ineffective against other solid tumors such as kidney and colon carcinomas. In addition, once a tumor becomes insensitive to a certain drug it will also show resistance to drugs whose structure and mechanism of action may be completely different (a phenomenon known as multi-drug resistance or MDR [13]). Drug sensitivity (inherent resistance) and the development of resistance during treatment are thought to be mediated by multiple mechanisms such as increased drug efflux, drug inactivation, mutations in the target protein and evasion of drug-induced damage or apoptosis [4,6]. 

For all these reasons, there is a constant demand for novel anti-cancer agents that could provide new treatment options by overcoming resistance mechanisms and, therefore, extending survival duration while minimizing toxicity and maintaining high quality of life.

In the past ten years, efforts have concentrated either on the development of mitosis-specific drugs that do not target tubulin but are inhibitors of key mitotic regulators such as aurora kinases, polo-like kinase I or the kinesin protein family (currently all in clinical development) [14], or on the discovery of new MTIs that, due to novel chemical properties, can overcome MDR induced by the classical MTIs.

Epothilones [15] and halichondrins [16] are examples of novel MTIs. Agents from both classes of compounds have shown very promising results in *in vitro* and pre-clinical studies in taxane-resistant cells and are in early stages of clinical development [14,17,18].

Here we characterize the mode of action of 4SC-207, identified in a small molecule screen as a novel cytotoxic agent. 4SC-207 inhibits proliferation in many tumor cell lines, including multi-drug resistance cell lines such as HCT-15 and ACHN. We demonstrate that 4SC-207 targets tubulin and acts as a microtubule destabilizing agent. It inhibits microtubule growth *in vitro* and *in vivo* and promotes, in a dose dependent manner, a mitotic delay/arrest followed by aberrant divisions or apoptosis. Additionally, 4SC-207 inhibits tumor growth in taxane resistant xenograft models and displays very low toxicity against bone marrow cells.

## Material and Methods

### Ethic Statement

The xenograft models were conducted in accordance with the principles outlined in the Australian Code of Practice for the Care and Use of Animals for Scientific Purposes, 7th Edition, 2004 (National Health and Medical Research Council). The protocol was reviewed and approved by the Animal Ethics Committee of the University of Adelaide (Permit Number M45-2008: Subcutaneous human xenograft tumour models utilized for the development of new human anti-cancer therapies).

### Compounds

4SC-207 was kindly provided by 4SC AG. Chemical formula: C_19_H_18_N_4_O_3_S. IUPAC name: (*E*)-ethyl 3-cyano-2-(3-(pyridin-3-yl)acrylamido)-4,5-dihydrothieno[2,3-c]pyridine-6(7H)-carboxylate. Exact mass: 382.11. 4SC-207 (stock 25mM in DMSO) and nocodazole (Sigma, stock 1.6mM in DMSO) were used at nM concentrations on cells and at μM concentrations in the *in vitro* tubulin polymerization assay.

### Cells and cell culture

HeLa wt cells (purchased from ATCC), HeLa EB3-EGFP cells [19] and HeLa H2B-mCherry/mEGFP-α-tubulin cells [20] (a kind gift from Dr. Daniel Gerlich, IMBA, Vienna, Austria) were cultured in DMEM medium supplemented with 10% FBS and 1% antibiotics (all Gibco). HeLa H2B-mCherry/mEGFP-α-tubulin cells were additionally supplemented with 0.5mg/ml Geneticin (Sigma) and 0.5μg/ml Puromycin (Invivogen). For imaging, the medium was exchanged to CO_2_-independent medium supplemented with 10% FBS, 1% Glutamax and antibiotics (all Gibco). For synchronization, cells were treated for 18hrs with 2mM thymidine. 

### RKOp27 proliferation assay

The assay was initially performed at Nycomed, Germany, and repeated at the University of Konstanz. The anti-proliferative activity of the compound was assessed in subclones of human RKO cells [21] using the Alamar Blue viability assay. 4SC-207 was dissolved in DMSO and diluted in semi-logarithmic steps. The final DMSO concentration in the assay was 1%. RKO cells were seeded into 96-well plates with a density of 4 x 10^3^ cells/well for proliferating RKOp27 cells and 1.6 x 10^4^ cells/well for arrested RKOp27 cells. The medium for arrested RKOp27 cells contained 10µM ponasterone A for induction of p27Kip1. 4SC-207 was added 24hrs after cell seeding and incubated for another 72hrs at 37°C. Cell viability was determined by Alamar Blue reaction (Invitrogen). Resulting viabilities are expressed in % values. Emission values of DMSO treated cells were set to 100% and used to normalize emission values of treated cells.

### FACS analysis

FACS analysis on RKO cells [21] was performed at Nycomed, Germany. The cells were cultured in MEM medium supplemented with 1x NEAA, 10% FBS and 1% Pen/Strep (all PAA) at 37°C with 5% CO_2_. Cells were seeded in 12-well plates at a concentration of 2 x 10^5^ cells/well. 16hrs after seeding, cells were either treated with 100nM 4SC-207 or 0.1% DMSO and incubated for 24hrs. The cells were then trypsinized, collected by centrifugation, washed twice with PBS and resuspended in a final volume of 50µl PBS. Subsequently, the cells were fixed by adding dropwise 100% ice-cold methanol while vortexing and incubated over night at -20°C. Next, the cells were centrifuged, washed with ice-cold PBS, resuspended in a final volume of 100µl PBS containing 1mg/ml Ribonuclease A (Sigma-Aldrich) and incubated for 5min at RT. To stain the DNA, 10µl of Propidium iodide (PI, BD Biosciences) was added and incubated for 5min at RT. Single channel flow cytometry was carried out on a Becton Dickinson FACS Calibur instrument and the analysis of the data was performed with BD Cell Quest software.

### Live cell experiments

For low-resolution imaging, HeLa wt cells were seeded at a density of 7 x 10^4^ cells/ml on a 12-well plate, synchronized with thymidine and treated with compounds (nocodazole, 4SC-207 and DMSO) 2hrs after thymidine release. Cells were imaged with a LD-Plan-Neofluar 20x, 0.4 N.A. Korr air objective lens (Carl Zeiss, Jena) on a Visiscope Analyzer (Visitron) equipped with a Cool Snap ES^2^ CCD camera (Photometrics). 2D multi-location time-lapse sequences of fields of cells were recorded for 15 hrs, t=10min.

For high-resolution imaging, HeLa H2B-mCherry/mEGFP-α-tubulin cells were seeded into 8-well chambers (IBIDI) and imaged with a Plan Apochromat 63x, 1.4 N.A. oil objective lens (Carl Zeiss, Jena) on a Zeiss Cell Observer HS equipped with a spinning-disk confocal unit and a Axiocam MRm CCD camera. 3D multi-location time-lapse sequences of fields of cells were recorded for 12hrs. Typical exposure times were 40-100ms and 3 to 5z steps (2-3μm apart) were acquired every 10min.

### Immunofluorescence experiments

HeLa wt cells were grown on coverslips, synchronized with thymidine and treated with the drugs (as described above). 9-10hrs after thymidine release cells were fixed with glutaraldehyde as described in [22]. To visualize microtubules, we used mouse anti-α-tubulin (Sigma F2168) FITC conjugated at a 1:500 dilution, and to stain the chromosomes Hoechst 33342 at 1:1000 dilution (Sigma, stock concentration: 1mg/ml). Images were taken with a PlanApo N 60x, 1.42 N.A. oil objective lens (Olympus) on a DeltaVision system (Applied Precision) equipped with a CoolSnap HQ^2^ CCD camera (Photometrics). 

### Image processing and quantification

Immunofluorescence images were acquired as raw 12-bit images at identical exposure times. Stacks were deconvolved in SoftWorx 3.5.0 (Applied Precision) and processed in ImageJ (http://rsbweb.nih.gov/ij/). Images shown in the same panel have been identically scaled.

For quantification of high and low-resolution live-cell experiments, cells were manually counted and categorized, mitotic duration was measured and spindle morphology annotated. Videos S1 to S7 were prepared in ImageJ.

### In vitro and in vivo tubulin polymerization assays

The polymerization *in vitro* of pig brain tubulin (stock concentration of 100μM) was measured by changes in the optical density of tubulin solutions at 350nm using a temperature controlled UV-Visible Spectrophotometer Cary 100 Bio (Varian). Three-times recycled tubulin was diluted (final concentration 10μM) on ice in glutamate buffer (0.8M, pH 6.6) with MgCl_2_ 100μM. DMSO (1:100), nocodazole (at 0.25, 0.5 and 1μM) or 4SC-207 (at 0.5, 1 and 2μM) were added to the reaction mix and incubated at 30°C for 10min. The samples were then returned to ice, supplemented with GTP (0.4mM) and transferred to the quartz cuvettes (150μl). The polymerization reaction was started by raising the temperature of the sample holder from 15°C to 30°C over a 2-min period. Absorbance at 350nm was measured every 30s for 20min. 

For the *in vivo* tubulin polymerization assay HeLa wt cells were plated in 8-well chambers (IBIDI). At the start of the experiment the cells were put on ice for 30min (cold treatment to depolymerize the existing microtubule network). DMSO (1:1000), nocodazole (at 500nM) or 4SC-207 (at 500nM) were added to different wells and the cells were then transferred to a plate heated at 37°C. Cells were fixed immediately (0min) or after 5min at 37°C. Fixation and staining procedures were performed as described above.

### Measurement of microtubule growth speeds

To measure microtubule growth speeds in cells we performed the assay described in [19]. In brief, HeLa EB3-EGFP cells were seeded in channel chambers (IBIDI, Slide VI) at a density of 4 x 10^5^ cells/ml, treated with DMSO, nocodazole or 4SC-207 and imaged with a Plan Apochromat 100x, 1.4 N.A. oil objective lens (Carl Zeiss, Jena) on a Zeiss Cell Observer HS equipped with a spinning-disk confocal unit and a Evolve EMCCD camera (Photometrics). 2D time-lapse sequences of single cells were recorded for 1min, t=400ms. As described in [19], movies were pre-processed in ImageJ, and then microtubule tips, labeled with EB3-EGFP, were automatically tracked and microtubule growth speeds and lengths were calculated. 20 cells per condition from two independent experiments were imaged and analyzed.

### Measurement of growth inhibition

Compound profiling on a large panel of 50 different cell lines was performed at OncoLead, Germany. Cell lines were purchased from ATCC. Cells used for the studies had undergone less than 20 passages. The cell lines were grown in the appropriate media according to suppliers protocols in the presence of 100 U/ml penicillinG and 100μg/ml streptomycin and seeded into 96-well plates for the experiment. 24hrs after seeding, cells were treated with different concentrations of 4SC-207 (six ten-fold pre-dilutions in DMSO were further diluted in RPMI medium, resulting in a final DMSO concentration of 0.1%) and allowed to grow at 37°C for 72hrs in 5% CO_2_. At the time of compound addition a control plate was immediately processed for cell number quantifications at time zero and to calculate cytotoxicity. To end the treatment, cells were precipitated by addition of 10% TCA (Sigma-Aldrich), fixed and, following overnight incubation at 4°C, the plates were washed 3 times with 400μl deionized water. Cells were then stained with 100μl of a 0.4% w/v sulforhodamine B (SRB, Sigma-Aldrich) solution in 1% acetic acid for at least 10min and then washed 6 times with 1% acetic acid to remove unbound dye. After drying at RT, bound SRB was solubilized with 100μl of 10mM Tris base. Measurement of optical density was performed at 520nm on a Victor2 plate reader (Perkin Elmer).

Growth inhibition of 50% (GI_50_) is the drug concentration resulting in a 50% reduction in the net protein increase compared to control cells. It is calculated as 100×[(Ti−Tz)/(C−Tz)] = 50, where Tz is the absorbance at time zero (drug addition), Ti the absorbance at the end of the treatment and C the absorbance of control cells at the end of the treatment.

### CFU-GM assay

The assay was performed at ReachBio LLC (Seattle, USA). Clonogenic progenitors of human and mouse granulocyte-monocyte (CFU-GM) lineage were assessed in a semisolid methylcellulose-based medium (R&D Systems). Medium contained cytokines rhSCF (50ng/ml), rhIL-3 (10ng/ml) and rhGM-CSF (10ng/ml) for human cells and rmSCF (50ng/ml), rmIL-3 (10ng/ml) and rmIL-6 (10ng/ml) for mouse cells. Normal human bone marrow light density cells (lot #0090714) derived from normal bone marrow (Lonza, Maryland) and qualified at ReachBio (in terms of progenitor frequency), were stored at -152°C until required for the assay. Cells were thawed and diluted in 10ml of Iscove’s modified Dulbecco’s medium containing 10% FBS and washed by centrifugation. The supernatant was discarded and the cell re-suspended in a known volume of IMDM + 10% FBS. Cell count (3% glacial acetic acid) and viability assessment (trypan blue exclusion test) were performed for the bone marrow sample. Mouse femoral bone marrow cells derived from an 8 week old Balb/c mouse were qualified at ReachBio (in terms of progenitor frequency), and stored at -152°C. Cells were prepared as described for the human bone marrow cells. 

The cultures were set up in triplicate at cell density of 3 x 10^4^ cells per culture for each condition and left to grow for 14 days after addition of the compound. 4SC-207 was dissolved in DMSO and working stock solutions at 1000, 100, 10, 1, 0.1 and 0.01μM were added to the methylcellulose matrix at 1:1000 v/v, to achieve a final test concentrations of 1000, 100, 10, 1, 0.1 and 0.01nM. IC_50_ and IC_90_ values were determined using appropriate software and the Boltzman equation y= [(A1-A2)/1 + e(x-x0)/dx ] + A2, where A1 = the initial value (baseline response), A2 = 0 (maximum response), x0 = center (drug concentration that induces a halfway response between A1 and A2) and dx = slope of the curve at midpoint.

### HCT-15 Xenograft model

The HCT-15 xenograft model was performed at VivoPharm (Kenttown, Australia). The study was conducted in accordance with the principles outlined in the Australian Code of Practice for the Care and Use of Animals for Scientific Purposes, 7th Edition, 2004 (National Health and Medical Research Council). 

HCT-15 human colon tumor cells were sourced from ATCC (Rockville, MD, USA) and cultured in RPMI 1640 or DMEM cell culture medium, supplemented with 10% FBS, 1% Glutamax and 1% penicillin-streptomycin. The cells were harvested by trypsinization, washed twice in Hank’s buffer salt solution (HBSS) and counted. The cells were then re-suspended in HBSS:MatrigelTM (1:1, v/v) to a final concentration of 1 x 10^8^ cells/ml. HCT-15 cells (100μl with 1 x 10^7^ cells) were introduced through the skin into the dorsal flank of female SCID mice. Mice have been randomized into 5 groups at an average tumor volume of 100mm^3^. Body weights were recorded for all animals on the first treatment day (Day 0) and then three times a week. Tumor dimensions (length and diameter) were measured for all animals on the first treatment day (Day 0) and then three times a week. Animals were terminated if individual tumor volume reached 2000 mm^3^ or if animals did not recover from a loss of more than 15% in body weight. Animals were anaesthetized and euthanized by exsanguination via terminal cardiac bleed.

The experiment consisted of 5 groups (N=10): Vehicle Control (Phosphal 50PG:Purified Water (20:80, v/v); Group 1); 4SC-207 (60 or 120mg/kg; Groups 2 and 3, respectively), 5FU™ (75, then 70mg/kg; Group 4) or Taxol® (10mg/kg; Group 5). Treatments continued for three weeks. Vehicle Control and 4SC-207 were scheduled for administration three times weekly via oral gavage (p.o.) in a dosing volume of 10ml/kg. 5FU™ was scheduled for administration once weekly via intravenous injection (i.v.) in a dosing volume of 5ml/kg (75 on Day 0, then 70mg/kg on Day 7 and 14; three treatments in total). Taxol® was scheduled for administration three times weekly i.v. in a dosing volume of 10ml/kg (nine treatments in total). Vehicle Control (Group 1), 4SC-207 at 60mg/kg (Group 2) and Taxol® (Group 5) were administered as scheduled. The treatment period for 4SC-207 at 120mg/kg (Group 3) was extended for one additional week while 5FU™ was inadvertently administered to mice in Group 4 on Day 9. This group was terminated prior to scheduled treatment on Day 14 as a result of adverse reaction. For the evaluation of treatment results vehicle group was compared to Group2 (60mg/kg 4SC-207) and Group3 (120mg/kg 4SC-207). Every measurement day, starting with day 2 up to day 19, was tested for statistical significance using a two-sided Mann-Whitney U-test (p=0.05).

## Results

### Identification of 4SC-207

**Figure 1:**
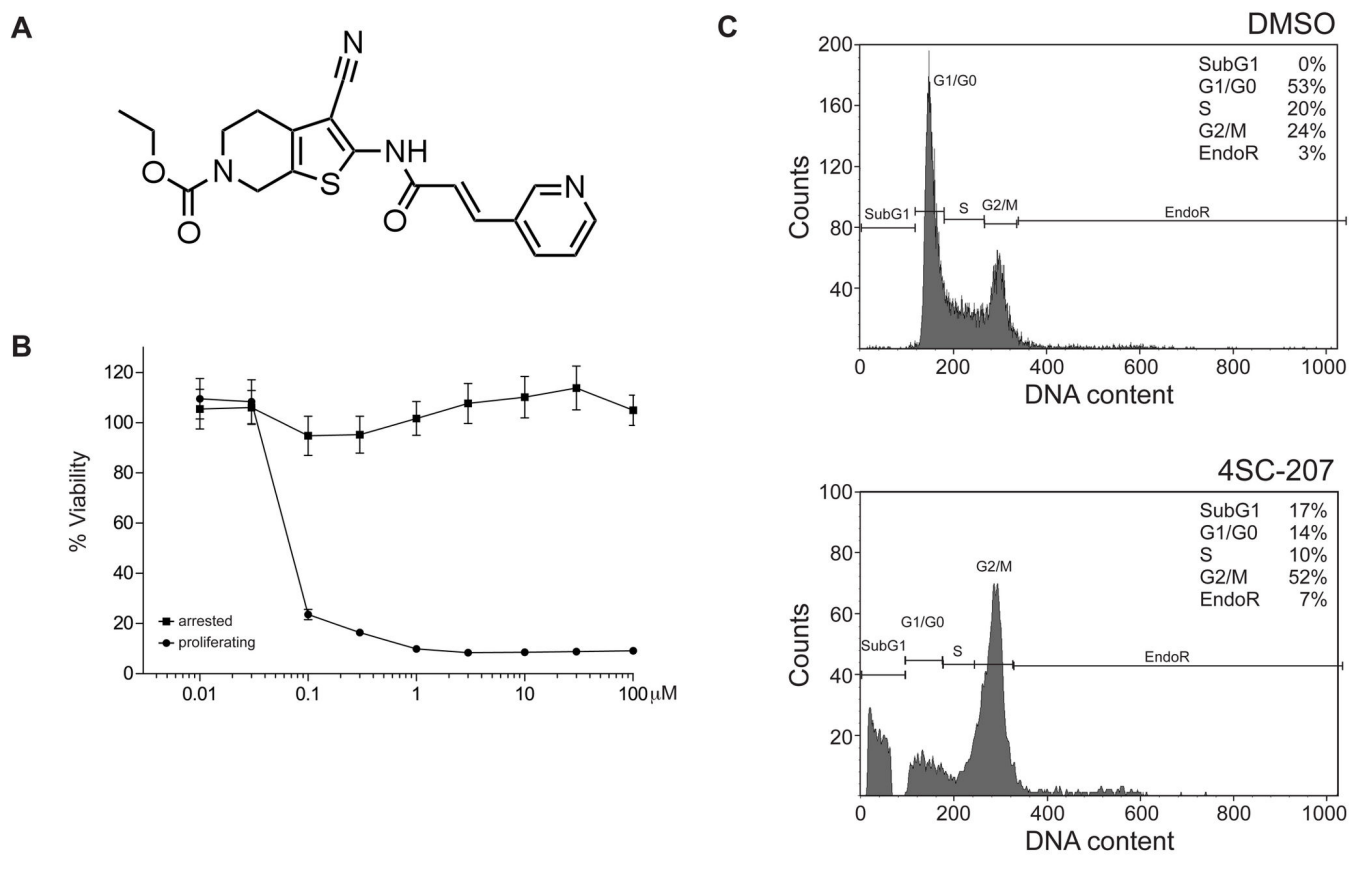
4SC-207 induces a G2/M arrest only in proliferating cells. (A) Structure of 4SC-207. (B) RKO cells arrested (■) or proliferating (●) were incubated for 72hrs with a range of 4SC-207 concentrations (from 10nM to 100μM) and cell viability was measured using the Alamar Blue assay (viability values were normalized to the DMSO-treated sample set to 100%). The mean of four independent experiments + SEM (standard error of the mean) is plotted in the graph. (C) Proliferating RKO cells were treated with 100nM 4SC-207 or 0.1% DMSO as a control. After 24hrs the cells were stained with PI and the cellular DNA content was determined by flow cytometry.

To identify new chemotypes, cytotoxic in a cell cycle dependent manner, a small molecule screen was set up using the genetically engineered tumor cell line RKOp27 [21]. Because RKOp27 cells can be arrested in G1 by the inducible overexpression of a p27Kip1 construct, the initial screen was set up to score phenotypic differences between proliferating and non-proliferating cells following compound treatments. 4SC-207 (Figure 1A) scored as a positive hit showing a cytotoxic effect only in proliferating but not arrested cells (data not shown). Titration of 4SC-207, as graphed in Figure 1B, confirmed that 4SC-207 specifically affects only proliferating cells over a broad range of concentrations, reducing the percentage of viable cells from 100% to 20% when added at or above a concentration of 100nM.

### 4SC-207 promotes a mitotic delay/arrest

To investigate the effect of 4SC-207 on the cell cycle, proliferating RKO cells were treated with the compound for 24hrs and then analyzed for their cell cycle state by flow cytometry. 4SC-207-treated cells showed a strong increase in the percentage of cells in G2/M phase (52% compared to 24% of DMSO-treated cells, Figure 1C) together with a decrease in the amount of cells in G1 and S phases (respectively 14% and 10% compared to 53% and 20% in control cells). Additionally, we observed the appearance of a subG1 population of cells (17%), suggesting the induction of apoptosis as a consequence of a prolonged G2/M arrest. 

**Figure 2:**
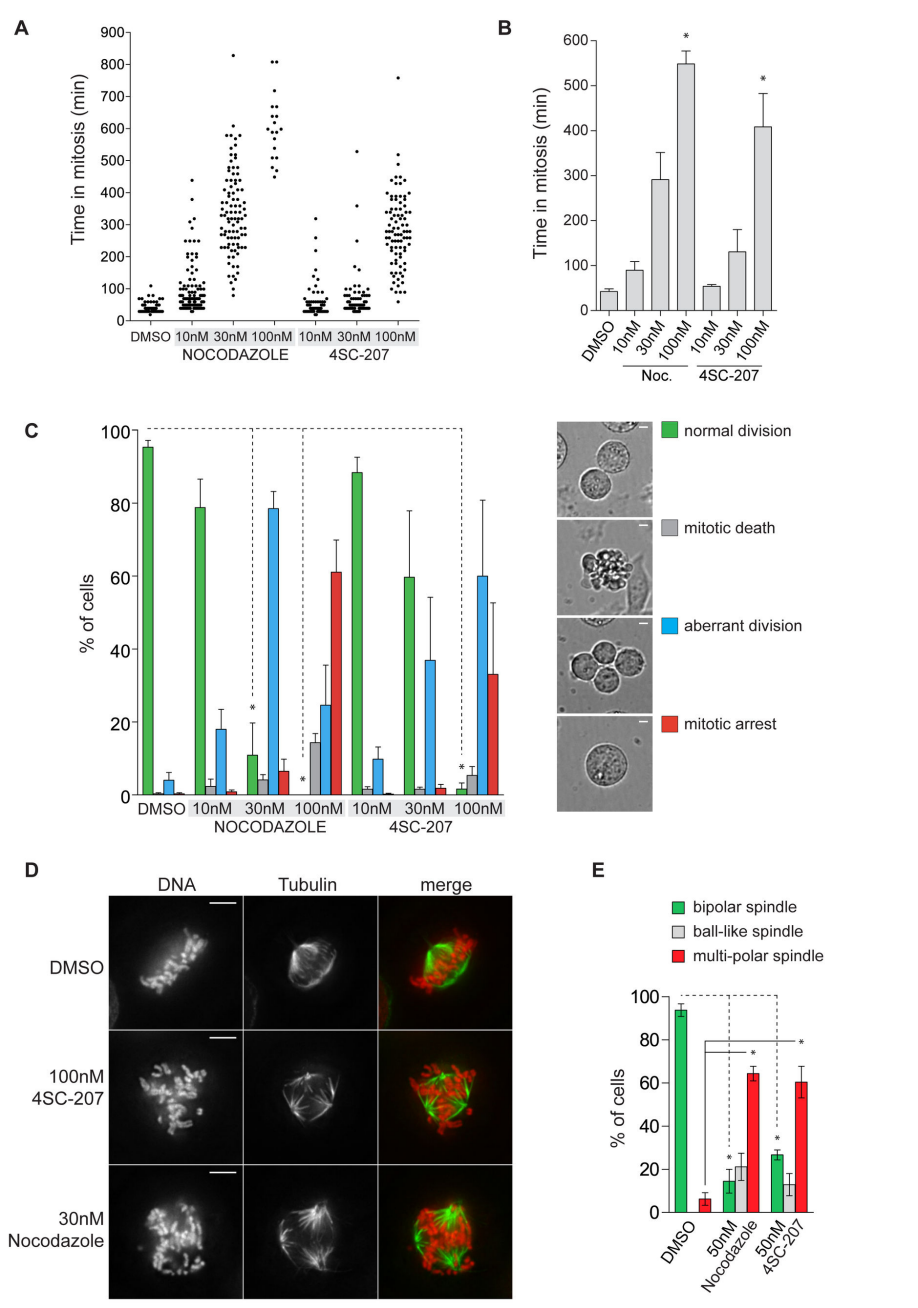
4SC-207 induces mitotic delay/arrest. Synchronized HeLa wt cells were treated at the end of S phase with 4SC-207, nocodazole (10, 30 and 100nM), or 0.1% DMSO and the first mitotic division was followed by time-lapse microscopy. (A) Scatter plot distributions of the mitotic durations for the different treatments from one representative experiment (for each condition 100 cells were counted and each dot represents one cell that completes mitosis in the indicated time). (B) Quantification of the mitotic durations from three independent experiments. Bars represent means + SEM (standard error of the mean). (C) Cells were categorized into four groups according to their fate after mitotic entry: cells that divided normally (‘normal division’, green), cells that died in mitosis (‘mitotic death’, grey), cells that divided into more than two daughter cells or failed to complete cytokinesis (‘aberrant division’, blue), and cells that arrested in mitosis (‘mitotic arrest’, red). Representative images for each category are displayed (scale bar 5μm). The mean of three independent experiments (100 cells per condition per experiment) + SEM is summarized in the bar chart. (D) HeLa wt cells were first thymidine synchronized then treated with 30nM nocodazole, 100nM 4SC-207 or 0.1% DMSO. 9hrs after thymidine release, cells were fixed and immuno-stained for α-tubulin (green) and DNA (Hoechst, red). Representative images are displayed as individual and merged channels (scale bar 5μm). (E) Quantification of spindle morphology from synchronized HeLa cells stably expressing H2B-mCherry/mEGFP-α-tubulin treated with 0.1% DMSO, 50nM nocodazole or 50nM 4SC-207. Spindles were categorized into three groups: bipolar, ball-like and multi-polar. The mean of three independent experiments + SEM is summarized in the bar chart (the total number of spindles for DMSO, nocodazole and 4SC-207 treated cells were, respectively, 138, 125 and 117). Statistical significance between compound treated and DMSO treated cells in panels (B), (C) and (E) was evaluated using the Student’s t-test and indicated with * (p<0.01 for panel (B) and (E), p<0.001 for panel (C)).

To better understand the nature of the G2/M block, live-cell imaging was performed on a synchronized population of HeLa wt cells. 4SC-207 or nocodazole, a well-known microtubule destabilizer that at low concentrations suppresses microtubule dynamics and induces a mitotic arrest [9], were added to cells 2hrs after thymidine release and the cells were observed for the following 20hrs. Approximately 9hrs after thymidine release, cells entered mitosis with similar kinetics independent of the treatment, suggesting that 4SC-207, like nocodazole, does not influence cell cycle timing prior to mitosis. For each condition, we scored 300 mitotic events from three independent experiments and documented whether i) cells were able to complete mitosis (in a normal or aberrant fashion), ii) cells were arrested in mitosis for the duration of the movie, iii) cells died during mitosis. Additionally, for those cells in the first category, we measured the time period from nuclear envelope breakdown (corresponding to cell rounding up) to anaphase onset and defined this as mitotic duration. As plotted in Figure 2A (a representative example) and in Figure 2B (the quantification of three independent repetitions) most DMSO treated cells completed mitosis with a median time of 45min while cells treated with 4SC-207 (or with nocodazole) showed a dose dependent increase in the time spent in mitosis. For example the mitotic duration of cells treated with 100nM 4SC-207 ranged from 100min to 550min (Figure 2A).

In Figure 2C, the mitotic events are categorized depending on their outcome and represented as % of the total events counted. At 100nM 4SC-207, all the cells that showed a mitotic delay in Figures 2A and 2B also exited mitosis in an aberrant fashion (blue bar, 60% of cells, only 2% divided normally as shown by the green bar). At the same compound concentration, the remaining cells arrested in mitosis (35%, red bar). We therefore conclude that 4SC-207 induces, in a dose dependent manner, aberrant divisions, following a prolonged mitosis, or mitotic arrest. At 100nM 4SC-207, all cells are affected by the compound.

We then investigated whether the observed mitotic phenotypes are a result of spindle assembly and chromosome alignment defects that have been well characterized for nocodazole [9]. We, therefore, performed immunofluorescence-staining experiments on HeLa wt cells synchronized with thymidine, treated with 4SC-207 or nocodazole, and fixed 9-10hrs after thymidine release, when most cells have entered mitosis. While in DMSO treated cells spindles were bipolar and all chromosomes were correctly aligned on the metaphase plate, in cells treated with 4SC-207 or nocodazole many chromosomes were misaligned (Figure 2D) and we observed that the degree of the alignment defect correlated with the appearance of multi-polar spindles. To follow the development of the phenotype rather than to capture only the end point event, we carried out high-resolution live-cell imaging experiments on HeLa cells stably expressing chromosome and microtubule markers (see Video S1 for control cells, Video S2 and S3 for 4SC-207 treated cells). In 4SC-207 treated samples, most cells assembled an initial bipolar spindle in which some chromosomes were correctly aligned while others remained close to one or both poles. Congression of the unaligned chromosomes was a very slow process, often not completed due to loss of spindle integrity and the appearance of extra poles (Video S2). The cells that completed mitosis, experienced in addition karyokinesis or cytokinesis defects that resulted in bi or multinucleated cells (Video S3). To evaluate whether multi-polarity is a characteristic phenotype of 4SC-207 (and nocodazole) treated cells, we counted over 100 mitotic events for each treatment from three independent live-cell imaging experiments and categorized spindles into three groups: bipolar, ball-like and multi-polar. We defined as ball-like those spindles that, compared to bi- or multi-polar spindles, showed a much higher degree of disorganization with no aligned chromosomes and disordered microtubule structures. As shown in Figure 2E, 60% of 4SC-207 (and nocodazole) treated cells resulted in multi-polar spindles while in control treated cells >90% of the spindles were bipolar. 

In summary, all the imaging data, from both live and fixed samples, suggest that 4SC-207 affects the process of chromosome congression: the similarities between the phenotypes of 4SC-207 and nocodazole treated cells (see Video S4 for nocodazole treatment) lead us to speculate that 4SC-207 may also directly target tubulin and affect microtubule dynamics.

### 4SC-207 inhibits tubulin polymerization and reduces microtubule growth speeds

**Figure 3:**
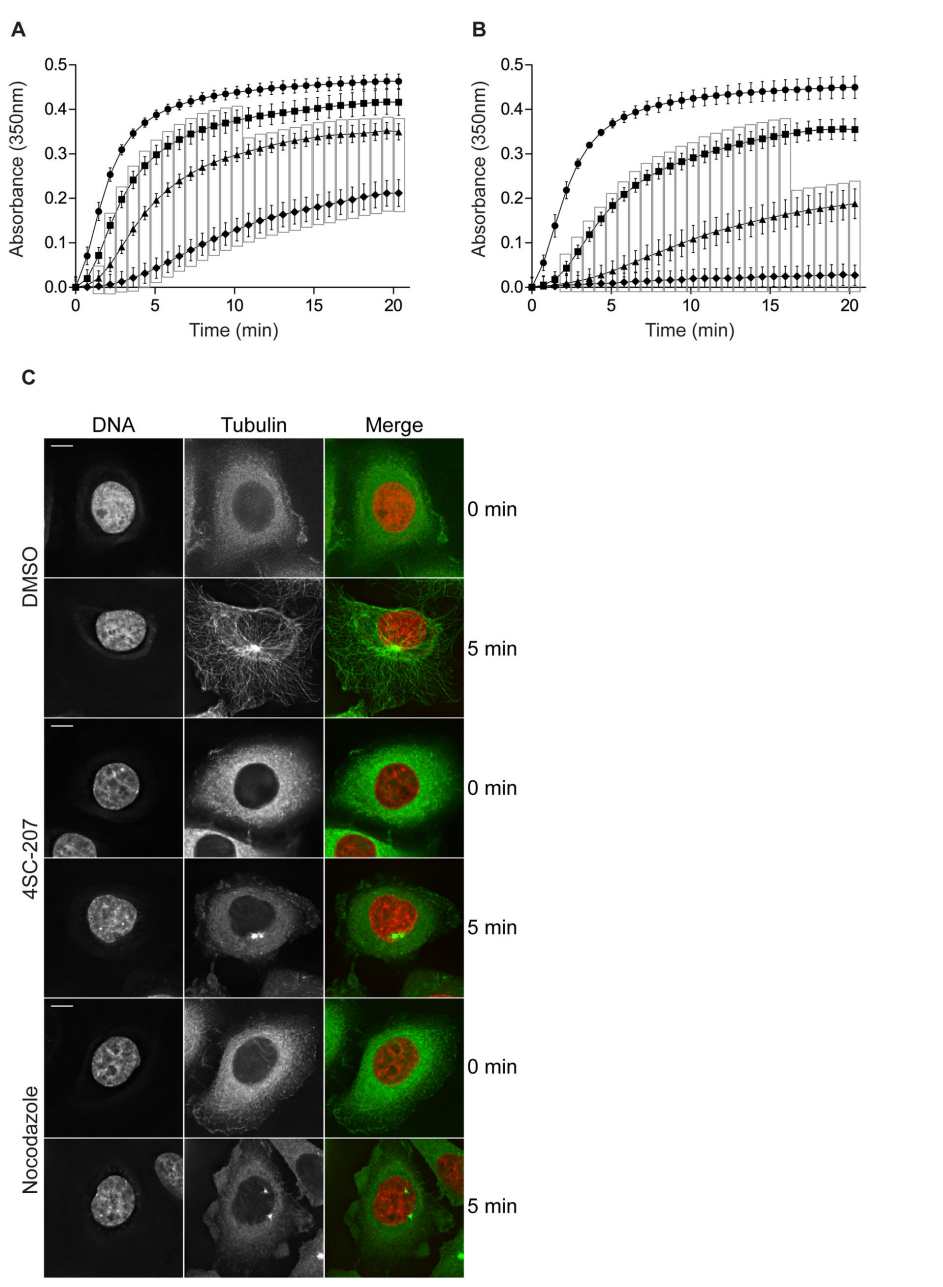
4SC-207 inhibits tubulin polymerization in vitro and in vivo. In vitro polymerization of tubulin, purified from pig brain, in the presence of 0μM (●), 0.25μM (■), 0.5μM (▲) and 1μM (♦) nocodazole (panel A) or 0μM (●), 0.5μM (■), 1μM (▲) and 2μM (♦) 4SC-207 (panel B). Results show the extent of tubulin polymerization as a function of time (absorbance at 350nm was monitored at 30s intervals). Each data point represents the mean of three independent experiments + SEM (standard error of the mean). For clarity, only even-data points were plotted. Statistical significance between data points of compound treated and DMSO treated samples was determined using the Student’s t-test and highlighted by a vertical rectangle (p<0.01). (C) Representative examples of HeLa wt cells following cold treatment (on ice), addition of 0.1% DMSO, 4SC-207 (500nM) or nocodazole (500nM) and fixed at 0min or 5min after transfer to 37°C. Cells were fixed and immuno-stained for α-tubulin (green) and DNA (Hoechst, red) (scale bar 5μm).

To test whether 4SC-207 is a MTI and whether it perturbs microtubule dynamics either by directly interfering with tubulin polymerization or indirectly by inhibiting the binding of microtubule associated proteins, we performed an *in vitro* microtubule polymerization assay [23]. DMSO, nocodazole or 4SC-207, were added to purified pig-brain tubulin solutions and polymerization was measured over a period of 20min. As shown in Figure 3, 4SC-207 (panel B), similarly to nocodazole (panel A), inhibits both rates and extent of tubulin polymerization in a dose-dependent manner. 

To verify whether 4SC-207 is capable of inhibiting tubulin polymerization also in cells, we carried out a microtubule regrowth assay following cold treatment. To do so, the existing microtubule network in cells was completely depolymerized by keeping the cells on ice for 30min. The cells were then re-transferred to 37°C in the presence of DMSO or high concentrations of 4SC-207 or nocodazole and fixed either immediately (0min) or 5min after transfer to 37°C. Cold treatment depolymerized all microtubules (Figure 3C, time point 0min) in all conditions, but while the microtubule network was completely re-established after 5min at 37°C in DMSO-treated cell, 4SC-207 and nocodazole strongly inhibited microtubule re-polymerization and after 5min at 37°C only short microtubule at the centrosomes were visible (Figure 3C, time point 5min).

We conclude, therefore, that 4SC-207 inhibits tubulin polymerization both *in vitro* and *in vivo*. 

**Figure 4:**
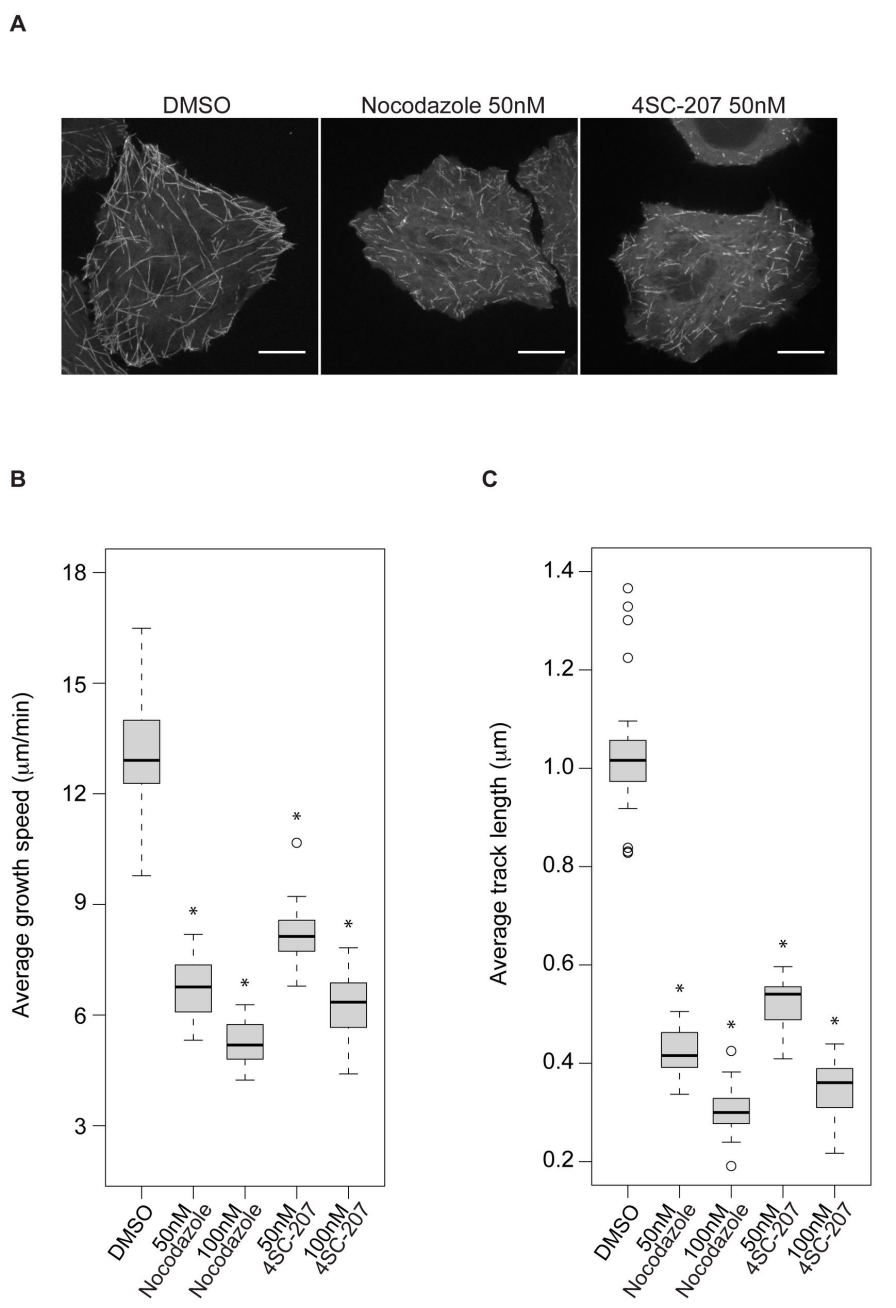
4SC-207 reduces microtubule growth rates and track lengths. (A) Time projections (the maximum intensity projections of all time points) of time-lapse sequences of interphase HeLa cells stably expressing EB3-EGFP (see Videos S5, S6 and S7) treated with 0.1% DMSO, 50nM nocodazole or 50nM 4SC-207 (scale bar 5μm). (B) Box plot representing the distributions of average growth speeds of microtubules in interphase HeLa cells stably expressing EB3-EGFP treated with 0.1% DMSO, nocodazole (50 and 100nM) or 4SC-207 (50 and 100nM). For each condition 20 cells were analyzed. (C) Box plot representing the distributions of average microtubule track lengths following the different treatments. For each condition 20 cells were analyzed. Statistical significance between compound treated and DMSO treated cells in panels (B) and (C) was evaluated using the Student’s t-test and indicated with * (p<0.001). In the boxes the median of the distributions is displayed as a thick line and the whiskers extend to Q3+1.5*IQR and Q1-1.5*IQR, were Q1=quartile1, Q3=quartile3, IQR=interquartile range (Q3-Q1). Samples that fall outside the boundaries are considered outliers (open circles).

Next we investigated the effect of 4SC-207 on microtubules in cells at concentrations that promote mitotic delay/arrest (as shown in Figure 2). To visualize microtubule dynamics we recorded high-resolution time-lapse sequences of interphase cells stably expressing EB3-EGFP, a microtubule plus-end marker (Figure 4A and Videos S5-7). By tracking the EB3 signal we could reconstitute most of the tracks of growing microtubules within the time frame of the movie. While in DMSO-treated cells microtubules grow with an average growth speed of ~13μm/min and microtubule lengths are on average ~1μm, 4SC-207, already at a concentration of 50nM, dramatically reduced microtubule growth speeds to ~8μm/min and lengths to ~0.5μm (Figures 4B and 4C). 

In summary, 4SC-207 is a novel microtubule destabilizer: at high concentration it inhibits microtubule polymerization while at low concentrations, by reducing microtubule growth speeds, it affects microtubule dynamics causing mitotic defects.

### 4SC-207 promotes growth inhibition in multi-drug resistance cancer cell lines

**Figure 5:**
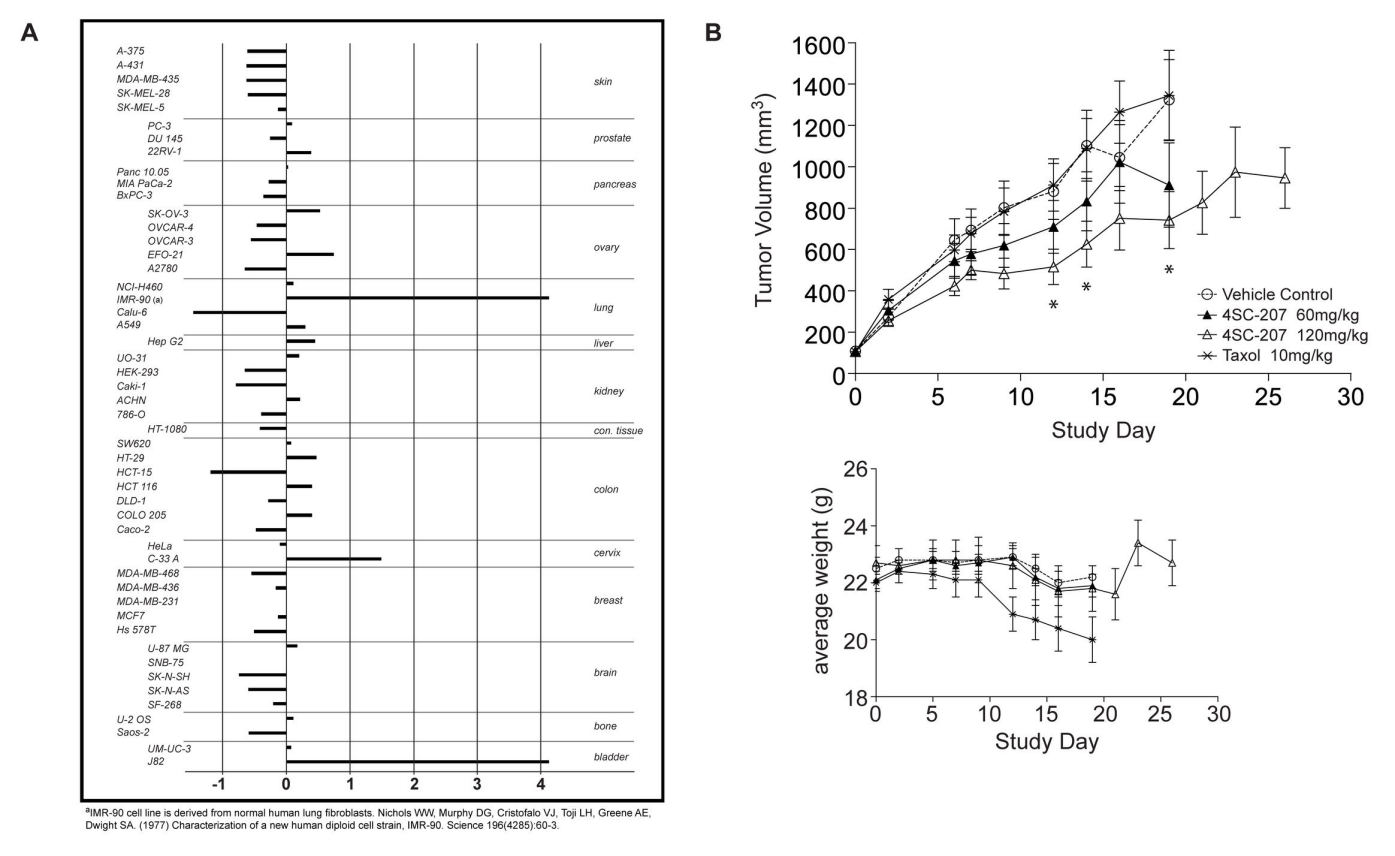
4SC-207 inhibits proliferation with an average GI_50_ of 11nM and promotes tumor growth reduction *in* *vivo*. (A) 4SC-207 was tested on a panel of 50 tumor cell lines from 14 different tissues and the GI_50_ values are represented as Z-scores, i.e. deviation of actual GI_50_ from the average GI_50_ divided by the standard deviation (SD). Negative Z-scores indicate higher sensitivity, positive Z-scores less sensitivity of cell lines towards the compound with respect to the mean GI_50_. The x-axis is displayed in log scale. (B) Female SCID mice bearing a HCT-15 colon cancer xenograft were treated with indicated concentrations of 4SC-207 (▲ and Δ), Taxol® (×) and control (○). Treatment was started at an average tumor volume of 100mm^3^ (Day 0) and continued for 3 weeks (treatment of 4SC-207 at 120mg/kg was extended for an additional week). Tumor volume and body weight were measured three times a week (see also Table 3). Significant reduction of tumor volume, indicated by * (two-tailed Mann-Whitney U-test; significance level p<0.05), was observed for treatment Group 3 (120mg/kg 4SC-207) on Day 12 (p=0.048), Day 14 (p=0.031) and Day 19 (p=0.025).

To verify whether the anti-proliferative activity that we observed in RKO and HeLa cells is a general characteristic of 4SC-207, we tested the compound on a panel of 50 different tumor cell lines at 6 different concentrations. Interestingly, 4SC-207 was active in nearly all cell lines with an average GI_50_ of 11nM. 29 cell lines (from 12 of the 14 different tissues tested) showed a GI_50_ <11nM (Figure 5A), suggesting indeed that 4SC-207 is a potent anti-proliferative agent active on a broad range of tumor cells. Amongst the cell lines that showed a very good response to 4SC-207 there were also known multi-drug resistant cell lines, such as DLD-1, HCT-15, ACHN and UO-31 [24]. For these cells lines and in addition for A2780AD, OVXL 899L and RXF 486L cell lines, not included in the initial screening panel, we compared the GI_50_ values of 4SC-207 with those of paclitaxel and vincristine (Table 1). 4SC-207 showed a stronger anti-proliferative activity in all cells tested, suggesting that tumor cells that have developed resistance to paclitaxel and vincristine would not show cross-resistance to 4SC-207.

**Table 1:** Growth inhibition of multi-drug resistant cell lines by 4SC-207, Paclitaxel and Vincristine.

	GI_50_ (nM)		
Cell line	4SC-207	Paclitaxel	Vincristine
DLD-1	6^a^	34	n.d.
HCT-15	9^a^	275	155
ACHN	16^a^	33	n.d.
UO-31	16^a^	28	n.d.
A2780AD	55	>1000	>1000
OVXL 899L	7	>300	n.d.
RXF 486L	15	>300	n.d.

^a^ Growth inhibition values were taken from Figure 4A

### 4SC-207, in pre-clinical studies, shows anti-cancer activity combined with low propensity to hematological toxicities

To evaluate the risk of induction of hematological side effects, we tested if bone marrow progenitor cells were sensitive to 4SC-207 to the same extent as proliferating cancer cells. To do so, we performed a granulocyte-monocyte colony formation unit assay (CFU-GM) on mouse and human bone marrow derived cells. The results (Table 2) were compared to published IC_50_ and IC_90_ values for vincristine and paclitaxel [25]. Contrary to expectations, mouse and human bone marrow cells can tolerate dosages of 4SC-207 that are 50 to 100-fold higher than those that induce proliferation defects in tumor cells (as shown in Figure 5A). 

**Table 2:** IC_50_ and IC_90_ values for Vincristine, Paclitaxel and 4SC-207 from mouse and human bone marrow CFU-GM assays.

	Vincristine^a^	Paclitaxel^a^	4SC-207
Mouse CFU-GM^b^			
IC_50_ (nM)	12	11	454
IC_90_ (nM)	30	27	950
Human CFU-GM^b^			
IC_50_ (nM)	1.2	4.7	490
IC_90_ (nM)	3	9	920

^a^ Kurtzberg LS, Roth SD, Bagley RG, Rouleau C, Yao M, Crawford JL, et al. Bone marrow CFU-GM and human tumor xenograft efficacy of three tubulin binding agents. Cancer Chemother Pharmacol2009 Oct;64(5):1029-38.

^b^ Refer to “Material and Methods” for assay description and IC_50_/IC_90_ calculation.

To test *in vivo* efficacy of 4SC-207 we chose the HCT-15 human tumor xenograft model, known to be resistant to taxol treatment [26]. Treatment with 60mg/kg or 120mg/kg of 4SC-207 resulted in a reduction in tumor growth, in a dose dependent manner, without evidence of toxicity based on body weight measurements (Figure 5B and Table 3). Statistically significant tumor reduction was observed for treatment with 120mg/kg 4SC-207 at day 12 (p=0.048) day 14 (p=0.031) and day 19 (p=0.025). On the contrary, as expected, treatment with 10mg/kg of Taxol had no effect on tumor growth but caused weight loss in the animals. 

**Table 3:** HCT-15 Xenograft model.

			**Tumor Response**		**Host Response**		
**Group**	**Treatment**	**Final Measurement Day**	**ΔT/ΔC (%)**	**Delta Tumor Volume ± SEM (mm^3^)**	**Delta Body Weight ± SEM (g)**	**% Delta Body Weight**	**Survival (Number Alive/ Total)**
1	Vehicle Control, *p.o.*	19	100.0	1283.6 ± 182.6	-0.2 ± 0.2	-1.0	9/10
2	4SC-207, 60mg/kg, p.o*.*	19	79.4	1019.8 ± 211.6	-0.2 ± 0.3	-1.0	8/10
3	4SC-207, 120mg/kg, p.o*.*	19	54.1	694.3 ± 134.8	-1.0 ± 0.8	-4.6	9/10
4	5FU™, 75 then 70mg/kg, i.v*.*	14^a^	-	-	-5.1 ± 0.4	-22.9	0/10
5	Taxol®, 10mg/kg, i.v*.*	19	105.6	1355.9 ± 188.7	-2.2 ± 0.5	-10.0	9/10

Tumor volume was calculated using the equation: V(mm^3^) = length x diameter^2^ x π /6.

Tumor variability was calculated as: Variability (%) = (SEM_Tumor Volume_ / TumorVolume_Mean_) x 100.

ΔT/ΔC (%) was calculated using the following equation: ΔT/ΔC % = (ΔVolume_Treatment_ / ΔVolume_Control_) x 100 where Δ = Change in volume from Day 0 to the final measurement day (or nominated day of interest).

^a^ This group was terminated prior to scheduled treatment on Day 14 as a result of adverse reaction (see “Material and Methods”).

In summary, 4SC-207 shows anti-proliferative activity also in the context of a tumor resistant to taxol treatment at a concentration that would not promote bone marrow toxicities.

## Discussion

Although taxanes and vinca alkaloids are highly effective anti-cancer agents and are extensively used in standard first-line chemotherapy, the development of resistance often renders them useless, in the context of secondary or metastatic cancers. For this reason there is a strong need for new chemotherapeutic agents that could provide effective treatment in second-line therapy, or whenever classical MTIs fail. 

Here we identify 4SC-207 that could respond to such demands. 4SC-207 belongs to the chemical class of tetrahydrothieno pyridines and so far this compound class had not been described to have anti-mitotic or microtubule inhibiting activities.

We demonstrate that 4SC-207 inhibits tubulin polymerization *in vivo*. The target is tubulin as confirmed by the *in vitro* tubulin polymerization assay where 4SC-207 inhibits in a dose dependent manner polymerization of purified pig-brain tubulin. Low doses of 4SC-207 (as shown for known MTIs [19]) strongly reduce the rate and extent of microtubule growth. Consistent with the accepted idea that MTIs induce mitotic defects as a result of suppressed microtubule dynamics [1], it is not surprising that cells treated with 4SC-207 are delayed in mitosis, unable to complete chromosome alignment or maintain a formed metaphase plate. Additionally, in many cells, spindles were disorganized and unstable due to the appearance of extra poles. Low doses of destabilizers have been shown to promote fragmentation of centrosomal material [9] or the formation of extra nucleation sites [27]. In both 4SC-207 and nocodazole treated cells, these extra poles were not *bona fide* centrosomes as centriolar markers was not detected (data not shown) but we speculate that they could be extra nucleation sites that arise following perturbations in microtubule dynamics. 

Prolonged mitotic arrest has been associated with uncoordinated loss of chromatid cohesion or centriole disengagements events [28,29]. However this phenotype has never been observed in conditions where microtubule dynamics were affected (such as in the presence of MTIs), suggesting that microtubule forces are required to destabilize cohesion. In cells treated with 4SC-207, we observed neither chromosome scattering nor centriole disengagement suggesting indirectly that the compound is targeting microtubule dynamics and therefore impairing the microtubule forces required for both phenotypes. 

Recently, another two MTIs, ixabepilone and eribulin, have been approved by the US Food and Drug Administration for the treatment of metastatic breast cancers that become refractory to anthracyclines and taxanes [30,31]. While ixabepilone belongs to the class of epothilones and is a microtubule-stabilizing agent, eribulin is a derivative of the halichondrin family and is a microtubule destabilizer. Regardless of their mode of action, the reason why they were approved for treatment of metastatic tumors is because both MTIs are active in taxane-resistant tumors that have developed mutations in β-tubulin [32], or that overexpress the βIII-tubulin isotype, to which paclitaxel binds with reduced affinity [33]. 

We tested 4SC-207 on a panel of cells derived from 50 solid tumors (from 14 different tissues) and we show that 4SC-207 is active at low nM concentrations in most cells, suggesting high potency and a very broad spectrum of activity. More importantly, amongst the cells tested we observed that 4SC-207 was also active in multi-drug resistance cell lines. DLD-1, HCT-15, ACHN and UO-31 cell lines are all known to be taxane-resistant due to overexpression of drug efflux pumps such as the ABC transporter P-glycoprotein (PgP) [24]. This suggests that 4SC-207 is not a substrate of P-glycoprotein and therefore it has the potential to be active in multi-drug resistant tumors that acquire resistance due to overexpression of PgP pumps. Epothilones are also not affected by overexpression of P-glycoprotein, even though the level of susceptibility was shown to be influenced by the substitutions present on the core structure of ephothilones [15], while, on the contrary, eribulin showed decreased *in vitro* activity in PgP expressing cells [34]. 

The potential of 4SC-207 to overcome MDR was confirmed also in the context of a tumor, in a HCT-15 derived human xenograft model (also taxane-resistant), where we observed that 4SC-207 retains activity and, in a dose dependent manner, reduces tumor growth, whereas paclitaxel, just like the vehicle control, does not influence tumor growth. In addition paclitaxel treated animals experience weight loss, while effective concentrations of 4SC-207 do not affect the health of the animals. This suggests that the concentration at which 4SC-207 is active in the tumor is below the concentration that would promote toxic effects. 

The balance between activity and toxicity is often the reason why MTIs cannot be administered at the dose that would be effective, because of the occurrence of severe side effects. The most commonly associated toxicities are the result of perturbations of microtubules in non-malignant cells, such as neutrophils and neurons. Neutrophils are the first line of defense against infections and, therefore, reduction in their number (termed neutropenia) can predispose patients to severe infections that, if not life-threatening, would compromise the outcome of the chemotherapy. Neurons are, on the contrary, non-dividing cells but are characterized by a very articulate and extensive microtubule network that is essential for their function. Epothilones and halichondrins represent a step forward compared to the classical MTIs because, together with the ability to overcome resistance, they also present a more manageable side effect profile. Both compounds are associated with neutropenia and neuropathy, however the incidence of febrile neutropenia (the highest degree of severity) and severe neuropathy are below 10% [15,35]. We do not have sufficient data to compare the side effect profile of 4SC-207 with that of eribulin and ixabepilone, however, the results of the CFU-GM assays are very encouraging as we show that mouse and human bone marrow derived cells can tolerate dosages of 4SC-207 that are 50 to 100-fold higher than the concentrations that induce proliferation defects in tumor cells, suggesting that neutropenia may not be dose limiting.

In summary, we identify 4SC-207 as a novel MTI. By combining strong anti-proliferative activity with reduced toxicity and the ability to overcome MDR, 4SC-207 shows great potential as an anti-cancer agent. Our data strongly support further investigations, especially concerning its mode of action and its activity in MDR cells.

## Supporting Information

Video S1 **Time-lapse sequence of a HeLa cells stably expressing H2B-mCherry/mEGFP-α-tubulin treated with 0.1% DMSO.** The cell was imaged on a spinning-disk confocal system with a Plan Apochromat 63x, 1.4 N.A., oil objective lens. 3D-stacks (5z slices, 2μm steps) were recorded for 12hrs with a time resolution of 10min. Selected time points were z-projected, cropped, contrasted and filtered in ImageJ.
(AVI)

Video S2 **Time-lapse sequence of a HeLa cells stably expressing H2B-mCherry/mEGFP-α-tubulin treated with 100nM 4SC-207.** Imaging and data preparation were identical as described for Video S1.
(AVI)

Video S3 **Time-lapse sequence of a HeLa cells stably expressing H2B-mCherry/mEGFP-α-tubulin treated with 100nM 4SC-207.** Imaging and data preparation were identical as described for Video S1.
(AVI)

Video S4 **Time-lapse sequence of a HeLa cells stably expressing H2B-mCherry/mEGFP-α-tubulin treated with 30nM nocodazole.** Imaging and data preparation were identical as described for Video S1.
(AVI)

Video S5 **2D time-lapse sequence of a HeLa cells stably expressing EB3-EGFP treated with 0.1% DMSO.** The cell was imaged on a spinning-disk confocal system with a Plan Apochromat 100x 1.4 N.A. oil objective lens for the duration of 1min with a time resolution of 400ms. The movie was filtered and contrasted in ImageJ.
(MOV)

Video S6 **2D time-lapse sequence of a HeLa cells stably expressing EB3-EGFP treated with 50nM nocodazole.** Imaging and data preparation were identical as described for Video S5.
(MOV)

Video S7 **2D time-lapse sequence of a HeLa cells stably expressing EB3-EGFP treated with 50nM 4SC-207.** Imaging and data preparation were identical as described for Video S5.
(MOV)
